# Dual-Fluoroscopy vs. Single-Fluoroscopy in Balloon Kyphoplasty: A Study of Efficiency and Safety

**DOI:** 10.3390/jcm13216608

**Published:** 2024-11-03

**Authors:** Roy Romem, Itzhak Engel, David Segal, Refael Behrbalk, David Schleifer, Jonathan EJ Koch, Nissim Ohana, Yuval Baruch

**Affiliations:** 1 Department of Orthopedic Surgery and Spine Surgery Unit, Meir Medical Center, Kfar Saba 4428164, Israel; 2 University of Pittsburgh Medical Center Children’s Hospital, Pittsburgh, PA 15224, USA

**Keywords:** vertebral compression fracture, kyphoplasty, fluoroscopy, imaging, radiation

## Abstract

**Background:** Vertebral compression fractures (VCFs) are the most prevalent type of osteoporotic fractures, often causing significant pain, morbidity, and mortality. Vertebral augmentation procedures like balloon kyphoplasty (BK) are effective in treating VCFs. These procedures are typically performed using a single fluoroscopy machine (SF) for anteroposterior (AP) and lateral views. We have implemented a dual-fluoroscopy (DF) technique to reduce procedure time and radiation exposure. The goal of this study was to determine whether dual-fluoroscopy could optimize surgical efficiency without compromising safety, offering a more effective alternative to traditional single-fluoroscopy methods. **Methods:** This retrospective study included 126 patients who underwent BK with either SF (n = 74, 58.7%) or DF (n = 52, 41.3%) between 2020 and 2024. We collected data on procedure duration per pedicle (PDPP), radiation exposure (reference air kerma and dose-area product [DAP]), and radiation duration. A sub-analysis of post-learning phase cases was performed. **Results:** A learning curve was identified for the first 24 cases and 15 cases using the SF technique and DF technique, respectively, which was followed by a stabilization in procedure duration per pedicle (Levene’s statistic = 10.623, *p* = 0.002 for SD difference, *p* < 0.001 for mean PDPP difference). After the completion of the learning phase for both techniques, the DF group demonstrated a significantly shorter PDPP (11.83 ± 4.3 vs. 14.03 ± 5.57 min, *p* = 0.049). No significant differences were found in radiation exposure, including radiation duration (*p* = 0.577), reference air kerma, or DAP. **Conclusions:** Dual-fluoroscopy significantly reduces procedure time after the learning curve is overcome, improving efficiency without increasing radiation exposure. This technique holds promise for optimizing kyphoplasty workflow and safety, supporting broader clinical adoption.

## 1. Introduction

Vertebral compression fractures (VCFs) are a common injury, particularly among elderly individuals with osteoporosis. In the U.S., up to 40% of people aged 80 and above are affected by VCFs [1]. These fractures contribute to significant pain, reduced mobility, and increased morbidity and mortality [2,3,4,5,6,7], creating a substantial burden on healthcare systems. In 2006, the inpatient cost for treating VCFs in the Medicare population was approximately $1.8 billion [7], and the financial burden is expected to grow by 50% between 2005 and 2025 due to the aging population and rising osteoporosis rates [8]. Although nonoperative treatments, such as analgesics, rest, and spinal bracing, may provide symptom relief for some patients, many continue to suffer from persistent pain and immobility, which can lead to secondary complications, including cardiovascular and respiratory issues [9]. For patients with refractory pain, vertebral augmentation procedures, including balloon kyphoplasty (BK) and vertebroplasty, offer more effective treatment options.

BK, which entails placing a balloon inside the collapsed vertebra, followed by the infusion of bone cement to restore vertebral height, has gained wide acceptance. Although vertebroplasty was once a subject of controversy [10], recent studies have demonstrated that BK offers significant advantages over nonoperative management, showing significant improvements in pain relief, reduced morbidity, and lower mortality rates [6,7,11,12,13]. Traditionally, these procedures are performed using a single fluoroscopic machine alternating between anteroposterior (AP) and lateral views. However, this method can be time-consuming and may increase radiation exposure for both the patient and surgical team. To address these limitations, we adopted a dual-fluoroscopy technique, utilizing one machine for the AP view and another for the lateral view. The aim of this study was to assess whether the dual-fluoroscopy technique could enhance the efficiency of BK compared to the traditional single-fluoroscopy method. Specifically, we sought to compare procedure duration and radiation exposure between the two techniques. By evaluating these outcomes, the study aims to determine whether dual-fluoroscopy optimizes workflow while maintaining patient and surgical team safety, with the potential for broader clinical adoption in high-demand surgical environments.

## 2. Materials and Methods

### 2.1. Study Design

This retrospective analysis reviewed data from patients who underwent BK at a single university hospital, between January 2020 and August 2024. The study compared the use of single-fluoroscopic imaging with dual-fluoroscopic imaging to assess its impact on procedure duration and radiation exposure. The primary outcome measure was the procedure duration (total, and per pedicle). Secondary outcome measures were radiation duration (total and per pedicle), and radiation exposure presented as reference air kerma (RAK), and dose-area product (DAP). Ethical approval was obtained from the Meir Medical Center IRB (institutional request number 0051-22-MMC, approval date: 20 July 2022), and patient confidentiality was maintained throughout the study.

### 2.2. Patient Population

The initial patient population comprised 196 individuals who underwent BK due to VCFs between January 2020 and August 2024. Seven patients were excluded due to missing data. Additionally, 70 cases were excluded because the lead surgeons were not implementing the dual-fluoroscopy technique, and had various experience levels that eliminated the possibility of assessing learning curves and perform standardization. In total, 126 patients were ultimately included, with 74 patients in the single-fluoroscopy group and 52 patients in the dual-fluoroscopy group. Demographic variables included patient age, sex, body mass index (BMI), and American Society of Anesthesiologists (ASA) score. Fracture characteristics were recorded by specific vertebral location and grouped into the following categories: thoracic, thoracolumbar junction, lumbar, and multiple vertebrae involvement. Additionally, the type of anesthesia used (general vs. sedation) was documented for each patient.

### 2.3. Data Collection

Data were extracted from electronic medical records and the hospital’s fluoroscopic monitoring systems. The following variables were recorded for each procedure:

Procedure Duration (Minutes): From Initial Needle Placement to Wound Closure
Radiation Duration (Seconds): Total Fluoroscopy Time
Radiation Exposure (RAK, Reference Air Kerma; Milligray, mGy): Total Radiation Dose Received by the Patient
Dose-Area Product (DAP; µGy·m^2^): Total Radiation Energy over the Exposed Area

### 2.4. Per-Pedicle Analysis for Standardized Comparisons

Since some patients underwent unipedicular and others bipedicular BK, and some procedures involved multiple vertebrae, a per-pedicle analysis was conducted to standardize comparisons. For each procedure, radiation exposure (RAK and DAP) and procedure duration were divided by the number of pedicles treated, allowing for more accurate comparison between the single- and dual-fluoroscopy groups.

### 2.5. Learning Curve Analysis

**Figure 1:**
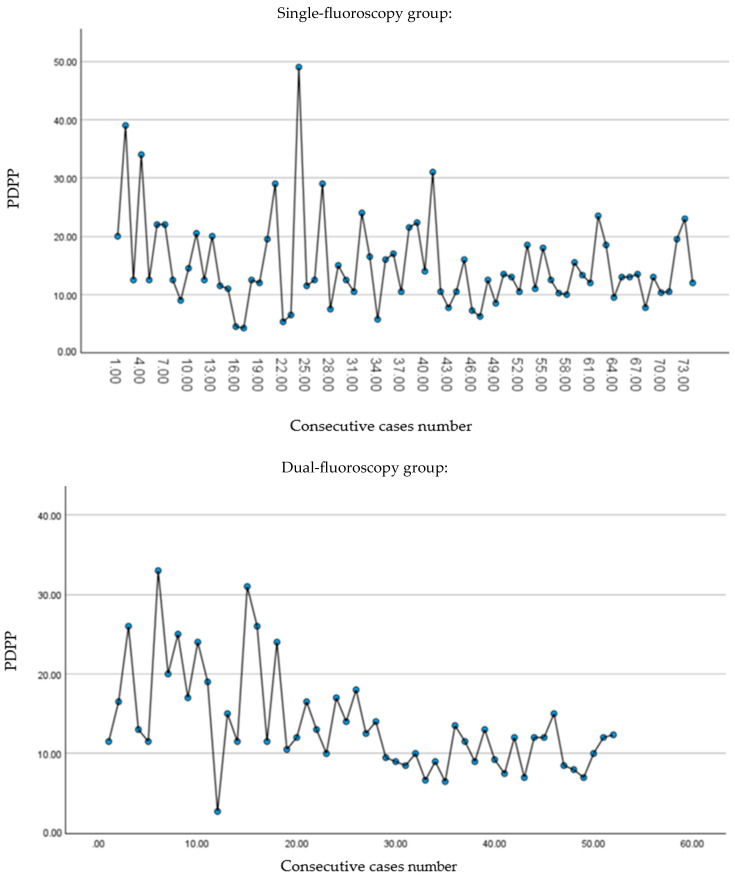
Scatter plot of PDPP for single-machine and dual-machine groups, plotted against consecutive case numbers. Abbreviations: PDPP, procedure duration per pedicle.

To assess the learning curve for both techniques, we plotted the procedure duration per pedicle (PDPP) over time, tracking consecutive cases (Figure 1). A trend of decreasing variability in PDPP was observed as surgeons gained experience, indicating an improvement in performance consistency. We applied Levene’s test for homogeneity of variance and manually divided the patient population into two groups: an early learning phase group and a plateau group. Various cut-off points were tested until we identified a statistically significant difference in the standard deviation (SD) of PDPP between the two groups. The stabilization of the SD at lower levels indicated reduced variability, implying improved predictability and reproducibility of the PDPP, signifying the completion of the learning phase. 

### 2.6. Statistical Analysis

Descriptive statistics were employed to outline patient demographics and procedural data. Categorical variables were presented with counts and percentages. Continuous variables, such as procedure duration, radiation duration, RAK and DAP, were reported as means and standard deviations. For the comparison between the single- and dual-fluoroscopy groups, independent *t*-tests were applied for normally distributed variables, while Mann–Whitney U tests were utilized for non-normally distributed variables. Analysis of categorical variables, such as anesthesia type and fracture location groups, was conducted using chi-square or Fisher’s exact tests, as appropriate. A *p*-value of less than 0.05 was established as the threshold for statistical significance. Data analysis was carried out using SPSS 28.0 software (Armonk, NY, USA, IBM Corp.).

### 2.7. Surgical Technique

**Figure 2:**
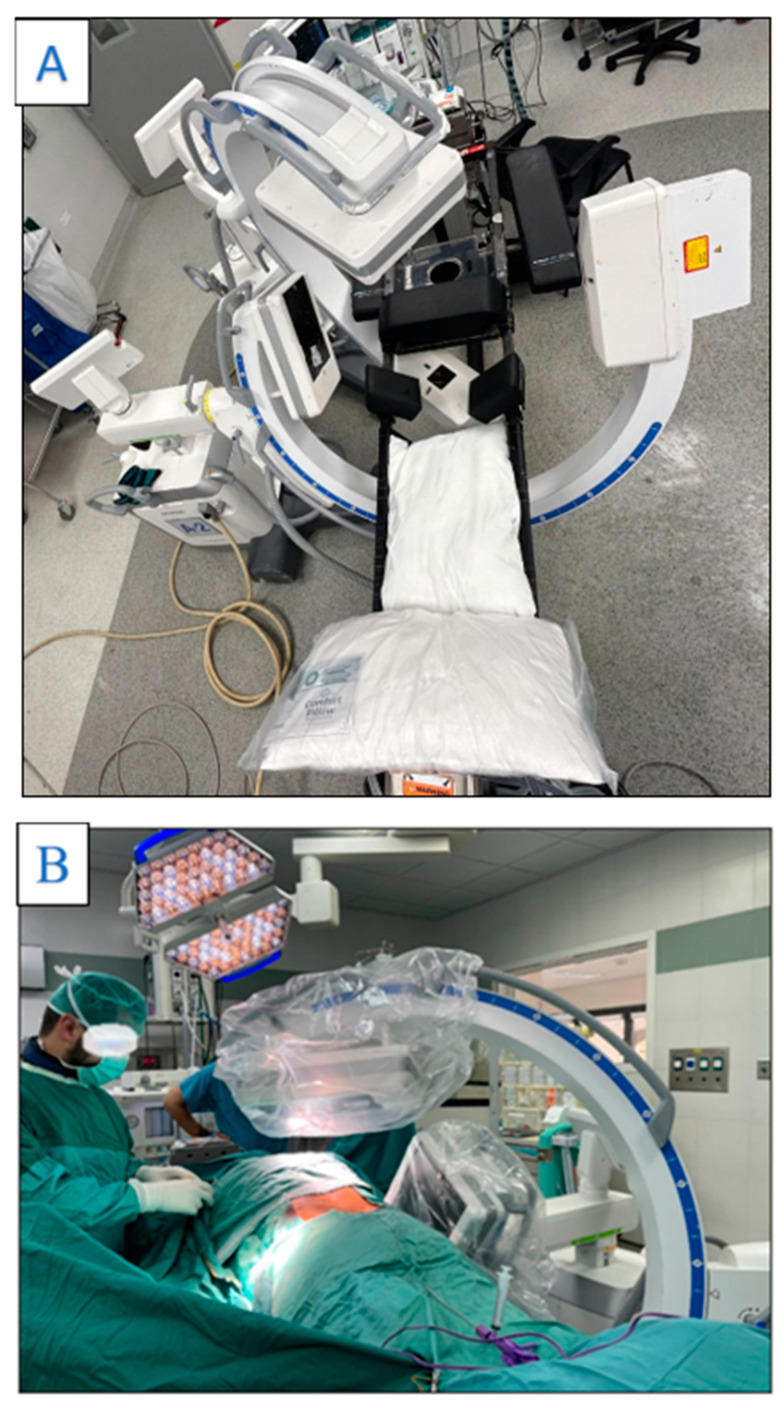
(**A**): Photograph depicting the C-arm setup in the dual-fluoroscopy group from a bird’s-eye view. One C-arm is positioned for anteroposterior imaging, and the other for lateral imaging, enabling simultaneous views during the balloon kyphoplasty procedure. (**B**): Demonstration of the surgeon’s position relative to the fluoroscopy machines, showing the optimal stance for controlling the procedure while minimizing radiation exposure.

Patients were anesthetized with either general anesthesia or local anesthesia with sedation, depending on their medical condition and cooperation. After anesthesia was administered, patients were placed in the prone position on an OSI table (Mizuho OSI, Union City, CA, USA). When implementing the dual-fluoroscopic technique, two separate C-arms were used (Siemens Cios, Erlangen, Germany)—one for anteroposterior (AP) and the other for lateral views. When using a single fluoroscopic machine, a C-arm of the same brand was rotated between AP and lateral views as needed (Figure 2). After identifying the index vertebra, a Jamshidi needle (CareFusion, San Diego, CA, USA) was inserted through the pedicle into the vertebral body using either a unipedicular or bipedicular approach, depending on the surgeon’s preference. Once in place, a collapsed balloon was inserted and inflated according to the manufacturer’s instructions (Confidence, Johnson & Johnson, New Brunswick, NJ, USA), followed by the injection of methyl methacrylate.

## 3. Results

### 3.1. Patients

The study encompassed a total of 126 patients, with 74 undergoing BK using single-fluoroscopy and 52 treated with dual-fluoroscopy. The mean age was 75.87 years (±9.32 SD), and 29.4% were males. The mean BMI was 26.55 ± 4.61 kg/m^2^. Most patients had an ASA score of 3 (57.1%), followed by ASA score of 2 (41.3%). The differences in the abovementioned characteristics between the two groups did not reach statistical significance (Table 1).

### 3.2. Surgical Variables

Most procedures (103, 81.7%) involved a single vertebra, while 23 procedures (18.3%) addressed two or more vertebrae. The fractures were primarily at the thoracolumbar junction (54%), followed by lumbar (33.3%) and thoracic (5.6%). Sedation was preferred and was used in 72.4% of cases. The unipedicular approach was employed in 24.6% of cases, while 75.4% used a bipedicular approach. The mean number of pedicles treated was 1.75, with no significant difference between the groups (*p* = 0.732). Full surgical and radiation data are summarized in Table 2.

### 3.3. Learning Curve

In the single-fluoroscopy group, the PDPP for the first 24 cases was 17.33 ± 11.13 min, while for cases 25 to 74 it dropped to 14.03 ± 5.57 min (*p* < 0.001, Levene’s statistic for SD difference = 11.863. *p* = 0.092 for the mean PDPP difference). Similarly, in the dual-fluoroscopy group, the PDPP for the first 15 cases was 18.45 ± 8.2 min, decreasing to 11.83 ± 4.3 minutes for cases 16 to 52 (Levene’s statistic = 10.263, *p* = 0.002 for SD difference. *p* < 0.001 for mean PDPP duration difference). No significant differences in mean PDPP, or radiation properties per pedicle, were observed between the early and plateau phases in either group. Thus, these measures were not added in the learning curve assessment. 

### 3.4. Sub-Analysis

A sub-analysis was performed, including only cases operated after the completion of the learning phase in each group (Table 3). In the dual-fluoroscopy group, the mean PDPP was significantly shorter compared to the single-fluoroscopy group (11.83 ± 4.3 vs. 14.03 ± 5.57 minutes per pedicle, *p* = 0.049). However, comparisons of radiation burden between the groups revealed no statistically significant disparities.

## 4. Discussion

As the global population ages, the prevalence of osteoporosis and VCFs continues to rise [1,8]. This trend has increased the demand for BK, a minimally invasive procedure to stabilize VCFs, highlighting the need for more efficient surgical solutions [6,13,14,15].

Fluoroscopy plays a vital role in BK, offering real-time imaging to ensure accurate placement of instruments and bone cement. With the growing number of osteoporosis-related fractures, there is a parallel increase in demand for fluoroscopy-guided surgeries, raising concerns about both surgical efficiency and radiation exposure. Prolonged surgeries not only increase the risk of complications, such as wound infections and cardiovascular issues, but also pose anesthesia-related risks [16,17,18]. Therefore, reducing surgical time is crucial, particularly in orthopedic procedures where radiation exposure is also a factor. One potential solution is modifying surgical techniques. Utilizing dual-fluoroscopy, for instance, not only shortens procedure times but also helps minimize radiation exposure for both patients and the surgical team.

Radiation exposure poses significant risks to healthcare staff, particularly orthopedic surgeons, who frequently work with fluoroscopy during surgeries. Prolonged exposure to ionizing radiation can lead to both acute and long-term health effects, including increased risks of developing cataracts, thyroid disease, and malignancies such as skin cancer and leukemia. Studies have shown that orthopedic surgeons are especially vulnerable, as they are often in close proximity to radiation sources during procedures such as spinal surgeries and fracture fixations [19,20,21,22].

Thoracic kyphoplasty is more challenging than lumbar procedures due to smaller pedicles, overlapping ribs, and reduced visibility of anatomical structures caused by interference from the lungs under fluoroscopic guidance [23,24,25]. These factors obscure landmarks, complicating pedicle localization and increasing the risk of procedural delays and adverse outcomes, such as misplacement of the Jamshidi needle and cement leakage. The need to toggle between AP and lateral views with a single fluoroscopy machine further disrupts workflow and precision. Dual-fluoroscopy overcomes these challenges by providing continuous AP and lateral views without machine adjustments, improving visualization and streamlining the procedure. This advantage is especially beneficial in multi-level interventions, where stable imaging eliminates repeated repositioning. With dual-fluoroscopy, multiple vertebrae can be treated more efficiently, reducing procedural time and minimizing risks. These benefits make dual-fluoroscopy particularly valuable in thoracic and multi-level kyphoplasty, where precision and efficiency are crucial for optimal outcomes.

An additional key advantage of using two fluoroscopic machines is that once both machines are positioned to provide simultaneous AP and lateral views, they remain stationary throughout the procedure. This allows the surgeon to control imaging via foot pedals, effectively reducing the need for a radiology technologist during the operation.

Our study successfully demonstrated that after mastering the dual-fluoroscopy technique, surgeons significantly reduced the PDPP compared to the single-fluoroscopy method. By carefully defining and accounting for the learning curve, we were able to show that once surgeons reached proficiency with the dual-fluoroscopy system, the efficiency of the procedure improved, resulting in consistently shorter operation times. This finding is particularly important, as shorter surgical times are associated with reduced anesthesia risks, lower patient morbidity, and enhanced operating room efficiency.

Beyond these immediate benefits, we believe that as surgeons refine their skills with the dual-fluoroscopy technique, procedure times could be further reduced, optimizing outcomes in high-volume surgical environments. Moreover, while our study did not show statistically significant improvements in radiation exposure metrics between the two groups, we anticipate that increased experience may lead to better radiation management, reducing exposure for both patients and the surgical team. 

These results highlight not only the current advantages of dual-fluoroscopy but also its long-term potential for enhancing both procedural efficiency and safety in spinal surgeries such as BK. Given the increasing demand for these procedures due to an aging population, the dual-fluoroscopy technique stands out as a promising advancement that warrants further exploration and adoption in clinical practice.

Our study had several limitations. First, its single-center design, which may limit the generalizability of the findings to other clinical settings with different practices or patient populations. Additionally, the relatively small sample size of 126 patients may restrict the statistical power of this study, and future studies with larger cohorts are needed to validate our results. The exclusion of 70 cases, in which the lead surgeons were not among those utilizing the dual-fluoroscopy technique, could introduce selection bias, as these excluded cases may differ significantly from those included. Another limitation is the involvement of only two surgeons, which could introduce bias in the learning curve analysis due to individual variations in technique and experience. Future research should incorporate multiple surgeons to more accurately assess the learning curve and reduce potential bias. Furthermore, this study did not include long-term patient outcomes, such as functional recovery or long-term complication rates, limiting our ability to fully assess the durability and long-term safety of the dual-fluoroscopy technique. Lastly, the study only evaluated short-term radiation exposure, leaving the long-term impact on both patients and healthcare staff unaddressed. Future studies should explore cumulative radiation exposure to provide a more comprehensive assessment of safety.

## 5. Conclusions

In conclusion, our study demonstrates that dual-fluoroscopy in balloon kyphoplasty reduces procedure duration per pedicle, significantly improving efficiency after the learning curve is mastered. With the growing demand for these procedures, dual-fluoroscopy provides a promising approach for optimizing surgical time and enhancing patient and radiation safety, making it a valuable method for broader adoption. Future studies should aim to validate these findings in larger, multi-center trials and assess long-term outcomes related to patient safety and radiation exposure.

## Figures and Tables

**Table 1:** Baseline characteristics of patients, categorized by single-fluoroscopy and dual-fluoroscopy groups.

	Single-Fluoroscopy Technique (74, 58.7%)	Dual-Fluoroscopy Technique (52, 41.3%)	Total (126, 100%)	*p*-Value
Age (y, SD)	75.41 ± 10.02	76.54 ± 8.27	75.87 ± 9.32	0.504
Male (n, %)	25 (33.8%)	12 (23.1%)	37 (29.4%)	0.235
BMI (kg/m^2^, SD)	26.15 ± 4.98	27.12 ± 4	26.55 ± 4.61	0.249
ASA score				0.09
1	0 (0%)	0 (0%)	0 (0%)	
2	26 (35.1%)	26 (50%)	52 (41.3%)	
3	47 (63.5%)	25 (48.1%)	72 (57.1%)	
4	1 (1.4%)	1 (1.9%)	2 (1.6%)	

Abbreviations: BMI, body mass index; ASA score, American Society of Anesthesiologists score.

**Table 2:** Surgical and radiation-related characteristics.

	Single-Fluoroscopy Technique (74, 58.7%)	Dual-Fluoroscopy Technique (52, 41.3%)	Total (126, 100%)	*p*-Value
Anesthesia (n, %)				0.089
Sedation	52 (70.3%)	44 (84.6%)	96 (76.2%)	
General	22 (29.7%)	8 (15.4%)	30 (23.8%)	
Region (n, %)				0.631
Thoracic	5 (6.8%)	2 (3.8%)	7 (5.6%)	
Thoracolumbar	38 (51.4%)	30 (57.7%)	68 (54%)	
Lumbar	24 (32.4%)	18 (34.6%)	42 (33.3%)	
Multiple	7 (9.5%)	2 (3.8%)	9 (7.1%)	
Vertebrae approached (mean, SD)	1.34 ± 0.688	1.13 ± 0.444	1.25 ± 0.606	0.064
Pedicles approached (mean, SD)	1.74 ± 0.44	1.77 ± 0.425	1.75 ± 0.432	0.741
Bipedicular technique (n, %)	55 (74.3%)	40 (76.9%)	95 (75.4%)	0.835
Procedure duration (minutes, SD)	29.26 ± 11.18	24.31 ± 8.2	27.21 ± 10.32	0.008
Radiation duration (seconds, SD)	77.64 ± 37.53	81.23 ± 32.19	79.13 ± 35.34	0.577
RAK (mGy, SD)	27.73 ± 18.47	36.29 ± 53.63	31.29 ± 37.29	0.201
DAP (µGy·m^2^, SD)	507.42 ± 335.92	510.57 ± 240.59	505.01 ± 299.2	0.914
Procedure duration per pedicle (mins, SD)	15.1 ± 7.89	13.74 ± 6.38	14.54 ± 7.31	0.305
Radiation duration per pedicle (secs, SD)	40.26 ± 27.03	43.96 ± 19.18	41.79 ± 24.09	0.399
RAK per pedicle (mGy, SD)	14.25 ± 12.67	18.44 ± 18.87	15.98 ± 15.6	0.139
DAP per pedicle (µGy·m^2^, SD)	260.86 ± 215.55	274.45 ± 159.87	266.47 ± 193.92	0.7

Abbreviations: RAK, reference air kerma; mGy, milligray; DAP, dose area product.

**Table 3:** Surgical and radiation-related characteristics after learning phase had been achieved.

	Single-Fluoroscopy Technique (n = 50)	Dual-Fluoroscopy Technique (n = 37)	Total (n = 87)	*p*-Value
Procedure duration per pedicle (mins, SD)	14.03 ± 5.57	11.83 ± 4.3	13.09 ± 5.1	0.049
Radiation duration per pedicle (secs, SD)	35.53 ± 19.11	38.67 ± 11.13	36.87 ± 16.2	0.374
RAK per pedicle (mGy, SD)	12.63 ± 8.01	16.71 ± 20.59	14.36 ± 14.77	0.204
DAP per pedicle (µGy·m^2^, SD)	240.63 ± 160.17	227.54 ± 95.41	235.07 ± 135.91	0.66

Abbreviations: RAK, reference air kerma; mGy, milligray; DAP, dose area product.

## Data Availability

Upon request, the authors will provide access to the raw data that substantiate the findings presented in this article.
